# High‐Throughput Screening for Accelerated Oxide Glass Discovery

**DOI:** 10.1002/advs.77590

**Published:** 2026-09-06

**Authors:** Igor Seibel‐Geraschenko, Josef Slowik, Franziska Scheffler, Alexis Duval, N. M. Anoop Krishnan, Lothar Wondraczek

**Affiliations:** ^1^ Otto Schott Institute of Materials Research Friedrich Schiller University Jena Jena Germany; ^2^ Center For Energy and Environmental Chemistry (CEEC) Friedrich Schiller University Jena Jena Germany; ^3^ Department of Civil Engineering Indian Institute of Technology Delhi New Delhi India; ^4^ Yardi School of Artificial Intelligence Indian Institute of Technology Delhi New Delhi India

**Keywords:** glass, high‐throughput screening, materials discovery, raman spectroscopy

## Abstract

Despite millennia of research and development, glass discovery remained largely reliant on manual, trial‐and‐error methodologies. A major impediment for accelerated glass discovery is its dependence on the processing conditions that is rarely documented, often complicating meaningful comparisons across independent studies. Here, a high‐throughput screening platform is presented that allows for up to one hundred cm^3^‐scale glass specimens to be produced per run through multicomponent stereolithographic printing, and be evaluated automatically via Raman spectroscopy without any sample preparation. The approach is demonstrated through its application to mixtures of representative yet chemically diverse glass systems, specifically, borosilicate, aluminosilicate and rare‐earth doped materials. The proposed strategy provides a scalable, accessible, systematic and quantitative framework for accelerated glass exploration with relevance across both academic and industrial research environments. Conventional laboratory synthesis throughput is exceeded by two orders of magnitude, while the reproducibility and compositional control that accelerated discovery demands are preserved.

## Graphical abstract

A high‐throughput screening platform is presented that allows for accelerating laboratory glass synthesis by a factor of 10^2^, using multicomponent stereolithographic printing. Automatic sample evaluation is achieved via Raman spectroscopy and similarity analysis without any sample preparation.

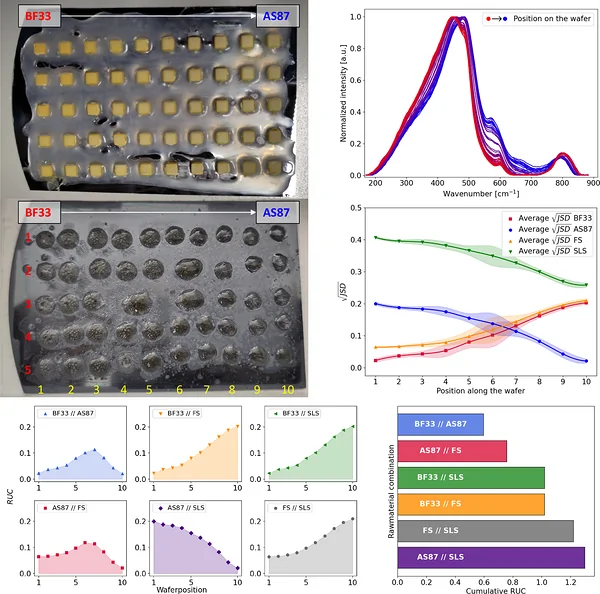

## 1 Introduction

High‐throughput screening (HTS) emerged in the 1990s, primarily in the sectors of pharmaceutical and biomedical research. Its core principles were delineated within a clear, conceptual framework by Broach and Thorner [1]. Notably, the primary aim of HTS, rather than identifying a compound meeting all criteria for a given application, is to survey a broad compositional space so as to detect lead candidates, or promising avenues warranting detailed follow‐up. To this end, analytical techniques are selected based on their scalability and compatibility with HTS workflows, rather than for exhaustive characterization. Over the ensuing decades, HTS has matured into a sophisticated methodology, contributing significantly to numerous discoveries, be it in the form of drug candidates, cosmetics or therapeutic agents [2, 3, 4]. More recently, the integration of these methodological advances, coupled with developments in artificial intelligence and large language models, has further enabled the extension of HTS to diverse scientific domains, such as the screening for functional ceramics [5] or metallic glass composites [6, 7]. However, HTS has been elusive in other domains. In particular, conventional glass discovery—despite its fundamental and recurrent role in societal progress—remains dominated by handicraft manual operations.

The applications of conventional (inorganic, non‐metallic) glasses span a wide spectrum of technologies, from everyday products (e.g., architectural or container glass) to advanced uses (e.g., telecommunication, high‐strength materials, photonics), as highlighted during the International Year of Glass (2022) declared by the United Nations [8]. While ongoing technological progress continues to escalate performance demands to meet societal objectives, including circular economy and carbon neutrality [9], research and development in glass is still grounded in manual, trial‐and‐error approaches. Traditional practices of laboratory glass discovery are inherently time‐, material‐, and energy‐intensive, but also often discard the (critical) influence of processing conditions on final material chemistries and properties [10, 11]. Alternative strategies, including modeling and simulation, are similarly constrained: they are inefficient for the exploration of large compositional spaces, often lack predictive accuracy when extrapolated beyond established case studies, and are usually ineffective for multi‐component systems (being the most relevant for real‐world application) [12, 13].

Effective implementation of HTS within the context of glass development requires the following key components: (1) a library of glass compositions with well‐characterized properties; (2) an autonomous system capable of combinatorial mixing of base materials in array‐based patterns; (3) a suitable carrier material for array‐based glass melting; (4) an automated characterization method requiring little to no sample preparation; and (5) a fully automated platform for data processing and mining of characterization outputs. Existing databases, such as SciGlass and Interglad, provide valuable resources by cataloging over 400,000 individual glass compositions and associated properties [13, 14]. In contrast, the most significant unmet challenge remains the realization of autonomous combinatorial mixing (requirement 2). Addressing this challenge requires overcoming three primary obstacles: first, the precise dispensing of base materials into discrete positions with well‐defined quantities; second, the efficient premixing of these materials to ensure compositional homogeneity while enabling short melting times, and third, a suitable characterization tool matching the rate of sample generation.

**FIGURE 1:**
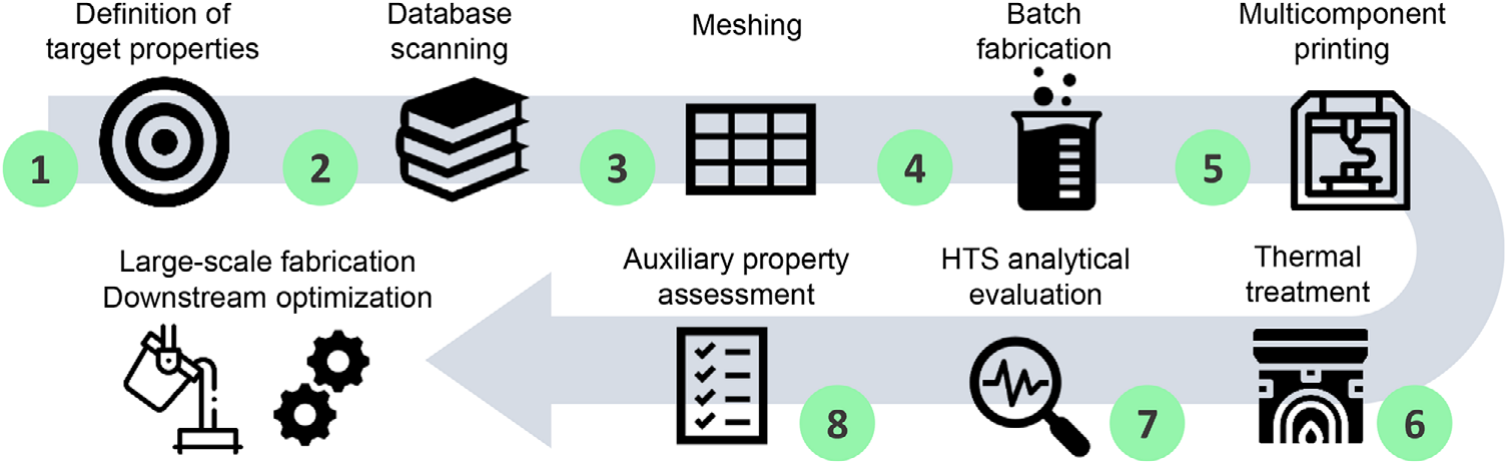
HTS workflow for glass discovery and experimental optimization.

In this report, we present a versatile, accessible HTS strategy for accelerating glass development, as shown in Figure 1. By using (i) a multicomponent printing approach, which enables the fabrication of arbitrary glass compositions by leveraging the high compositional flexibility of feedstock systems, and (ii) silicon wafers as carrier substrates, we achieve a production rate of 10^2^ samples per day, for cm‐scale samples—exceeding conventional laboratory synthesis (constrained by crucible usage and cleaning) by two orders of magnitude.

Recent progress in the additive manufacturing of glass has focused on either the fabrication of complex, transparent 3D architectures [15, 16, 17], or the expansion of sol‐gel processing routes [17, 18, 19]. Nevertheless, these approaches frequently limit the range of usable precursors, thereby constraining the accessible compositional space and posing challenges for HTS applications. We therefore focus on a universal precursor formulation for flexible powder loading, and use multicomponent printing for slurry admixture rather than the ultimate fabrication of intricate 3D structures.

Benefitting from wafer‐based processing, we furthermore developed and validated a quantitative Raman‐based platform capable of high‐throughput and automated material classification without prior sample preparation, benchmarked against representative glass standards. This approach demonstrates substantial potential for applications both in academic and industrial context, as exemplified by the presented use cases of adapting the dopant concentration in a photoluminescent glass, and adapting the glass transition temperature *T*_g_ across a series of aluminosilicate to borosilicate compositions.

## 2 Results

### 2.1 Print Quality and Process Validation

**FIGURE 2:**
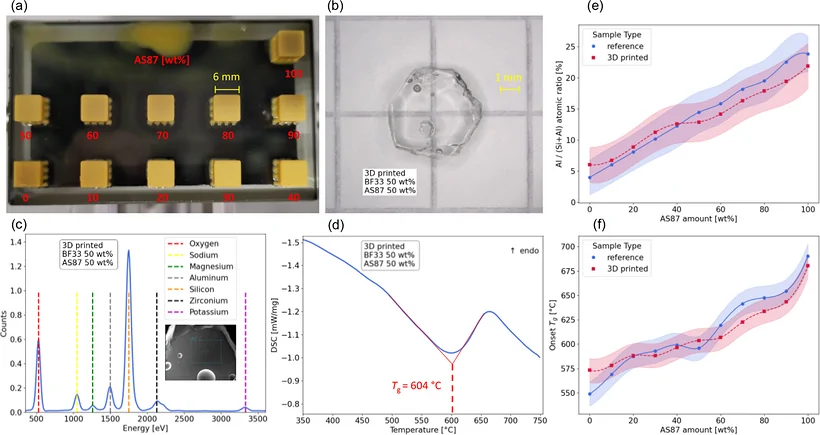
Sample preparation by component admixture using multi‐component 3D printing. (a) Printed specimens comprising mixtures of BF33 and AS87 with 10 wt.% compositional increments. (b) Photograph, (c) EDS spectrum, and (d) DSC scan of the printed, melted, and then polished 50 wt.% BF33 / 50 wt.% AS87 glass specimen. Derived compositional dependence of (e) the Al/(Si + Al) atomic ratio, and (f) the glass transition temperature (*T*_g_).

Prior to demonstrating HTS applied to glass science, the method was first validated at a smaller scale to assess print quality. To this end, two batches of eleven samples—comprising mixtures of BF33 and AS87 with 10 wt.% compositional increments—were prepared, either via a conventional glass synthesis route (serving as a reference, involving weighing, manual homogenization, and melting) or by first printing parallelopiped specimens (with dimensions of 6 × 6 × 3 mm^3^), as presented in Figure 2a, followed by melting; a representative resulting glass is displayed in Figure 2b. The final glasses are transparent and free of phase separation or crystallization, thereby highlighting the suitability of the multicomponent printing strategy for HTS implementation. Furthermore, the compositional evolution—reflected by the Al/(Si+Al) atomic ratio, increasing from 3% for BF33% to 25% for AS87—and the glass transition temperature *T*_g_ (shown in Figure 2e,f respectively), derived from individual EDS (Energy‐dispersive X‐ray spectroscopy, shown in Figure S1) spectra and DSC (differential scanning calorimetry) scans with representative examples illustrated in Figure 2c,d, reveal no discernible discrepancies between the printed and conventionally synthesized glass series. Only at the two extremes of the series (100 wt.% BF33 and 100 wt.% AS87), slight deviations are observed, though, still within the experimental error, which are ascribed to insufficient cleaning during the printing process when switching between slurries, resulting in minor cross‐contamination. Together, these results confirm the high printing fidelity, compositional homogeneity, and limited alkali volatilization of the proposed framework.

### 2.2 Raman‐Assisted Compositional Evaluation

**FIGURE 3:**
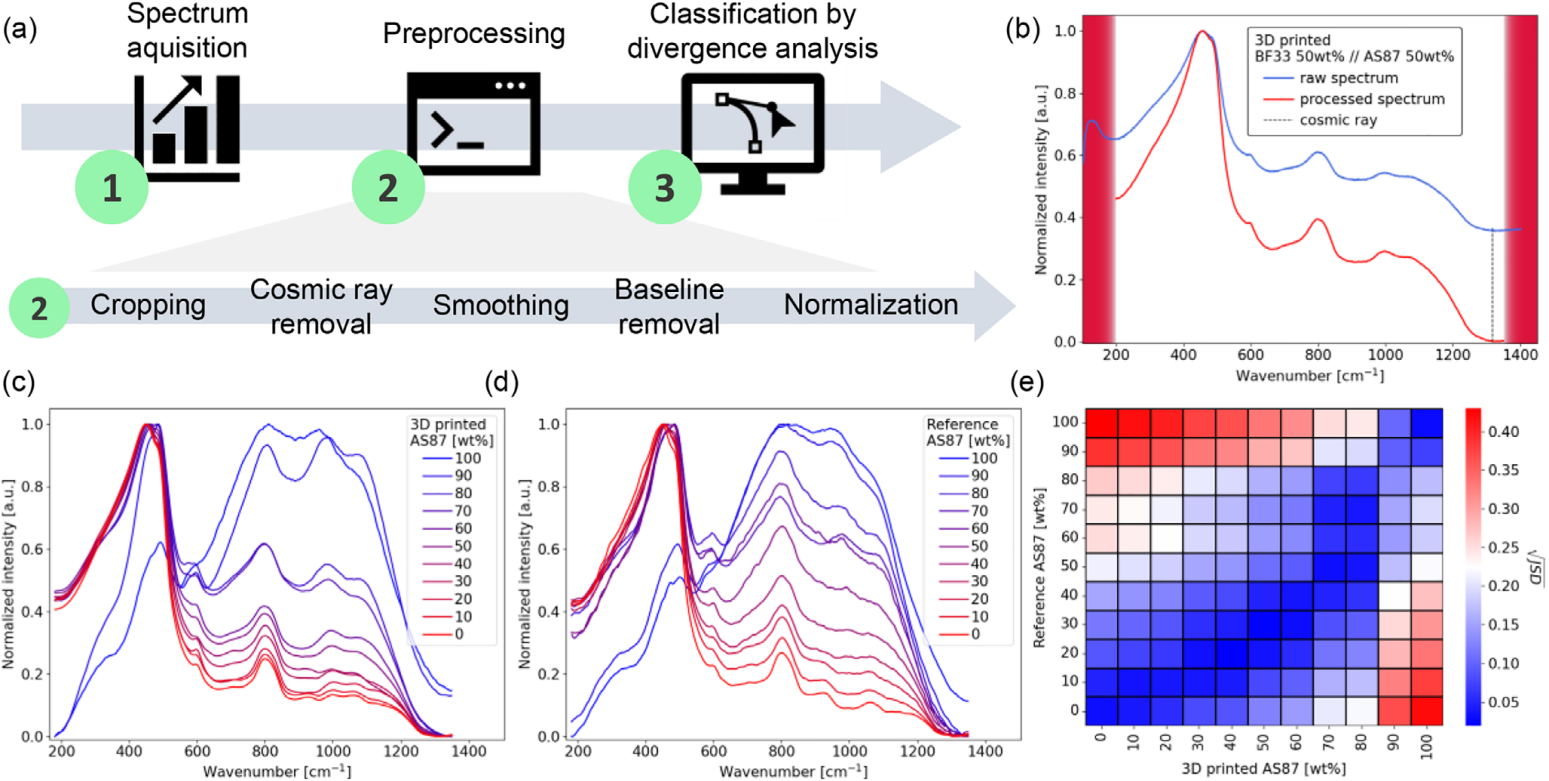
High‐throughput material classification by Raman spectroscopy. (a) Numerical workflow from spectra acquisition to distance calculation with (b) an example spectrum of the 50 wt.% BF33 / 50 wt.% AS87 specimen, normalized Raman spectra of (c) the printed, then melted specimens, and (d) the conventionally synthesized specimens. (e) Heatmap highlighting the square root of the Jensen‐Shannon divergence (JSD) when comparing the reference and the printed, then melted specimens.

EDS and DSC provide valuable physicochemical information on the obtained specimens; however, they present notable limitations preventing their use for HTS, the most significant being the requirement for sample preparation (e.g., cutting, polishing, grinding, and/or sputtering) [20]. In contrast, Raman spectroscopy—widely used in glass science and supported by well‐established catalogs of active vibrational modes—offers a suitable alternative. Indeed, in addition to providing a material signature and embedding quantitative structural insights in direct link to its chemical composition, Raman spectroscopy can be performed without any prior sample preparation through rapid measurements on the order of minutes, or even seconds [21, 22, 23]. Accordingly, the Raman spectra measurement and subsequent numerical procedure is shown in Figure 3a and demonstrated on the 3D‐printed 50 wt.% BF33 / 50 wt.% AS87 specimen in Figure 3b. All Raman spectra for both glass series (printed, and reference specimens) are shown in Figure 3c,d, respectively. For BF33‐rich samples, the most intense vibrational modes lie in the low‐frequency region between (200 and 650 cm^−1^), which vary with the degree of polymerization and represent inter tetrahedral linkage vibrations [24]. These contributions progressively decrease relatively with increasing AS87 content, accompanied by variations in the relative intensities of Q^n^ tetrahedral species in the high‐frequency region (900 and 1300 cm^−1^), reflecting changes in former‐to‐modifier ratios. Overall, the printed and reference series exhibit comparable Raman spectra, albeit the latter displays a more monotonous compositional dependence—which is ascribed to slight slurry cross contamination.

For a high‐throughput process involving tens to hundreds of individual samples, a manual, visual classification of Raman spectra becomes impractical; therefore, an automated, numerical comparison method is required. To this end, a dissimilarity metric was adopted. Within this framework, Raman spectra are treated as probability distributions representing the likelihood of observing a vibrational mode at a given wavenumber. The comparison of spectra from 3D‐printed specimens with references can therefore be formulated as an assessment of how consistent the observed distribution of vibrational modes is with a reference distribution. A commonly used measure for quantifying differences between probability distributions is the Kullback‐Leibler divergence (*D_KL_*) [25]. The discrete form writes:

(1) $$D_{KL}\left(P\;||\;Q\right)=\sum_{x\in X}\;p\left(x\right)\;\cdot \;log\frac{p\left(x\right)}{q\left(x\right)}$$

where P and Q represent two Raman spectra, X denotes the wavenumber array, and p (x) and q (x) correspond to the intensity values at wavenumber x. It is noteworthy that, after area normalization of the spectra, these values can be interpreted as the probability of observing a vibrational mode at a given wavenumber x. While the Kullback–Leibler divergence exhibits several convenient properties—such as being non‐negative and equal to zero only when the two distributions are identical, and quantifying the inefficiency of assuming distribution Q when the true distribution is P [26], a critical limitation in this context is its asymmetry. In other words, comparing spectrum P to Q yields a different result than comparing Q to P. To address this limitation, a symmetric metric derived from *D_KL_*—the Jensen‐Shannon divergence (JSD)—was employed [27], thereby providing a symmetric measure of similarity between two probability distributions:

(2) $$JSD\left(P\;||\;Q\right)=\frac{1}{2}\;D_{KL}\;\left(P\;||\;M\right)+\frac{1}{2}\;D_{KL}\;\left(Q\;||\;M\right)$$

where $M=\frac{1}{2}\;\left(P+Q\right)$ represents the mixture distribution of P and Q. Further, taking the square root of the JSD yields the corresponding distance metric (for which lower values indicate a higher probability of correspondence between the two spectra), thus allowing its use to construct a distance landscape comparing printed samples with reference spectra. This landscape is subsequently used for high‐throughput classification, enabling rapid assignment of a recorded spectrum to the most probable database candidate, thus acting as a consistency check for high‐throughput synthesis as well as for identifying structural similarity when screening for unknown formulations.

The resulting distances for the present case study are represented as a heatmap in Figure 3e. Here, it is evident that printed specimens with larger AS87 contents correspond most closely to reference samples with similarly large AS87 fractions, in direct agreement with the trends previously discussed results utilizing more preparation‐intensive techniques such as EDS and DSC. Nevertheless, in an ideal case, the heatmap would exhibit a strictly linear compositional dependence of the minimal distances along its diagonal, effectively dividing the matrix into two symmetric halves. The minor deviations observed from this behavior can be attributed to several factors. First, even small compositional variations (below the detection limit of EDS), including alkali or boron volatilization, can significantly alter the final Raman spectrum. Second, Raman spectra of AS87‐rich glasses were found to be highly sensitive to thermal history—effectively emphasizing the relevance of HTS approaches for obtaining and subsequently comparing materials processed under identical thermal conditions. Finally, the reference spectra themselves exhibit a degree of self‐similarity (see Figure S2); for instance, the 60 wt.% and 70 wt.% AS87 reference spectra are nearly overlapping. As a result, the heatmap exhibits a diffuse, asymmetrical gradient of minimal distances along the compositional series. Despite these effects, the overall gradient remains sufficiently clear to enable rapid and reliable identification of printed samples relative to reference compositions, demonstrating the robustness of this analysis strategy for evaluating printing performance and establishing the foundations for its application in an HTS context.

### 2.3 Extension to High‐Throughput Glass Discovery

**FIGURE 4:**
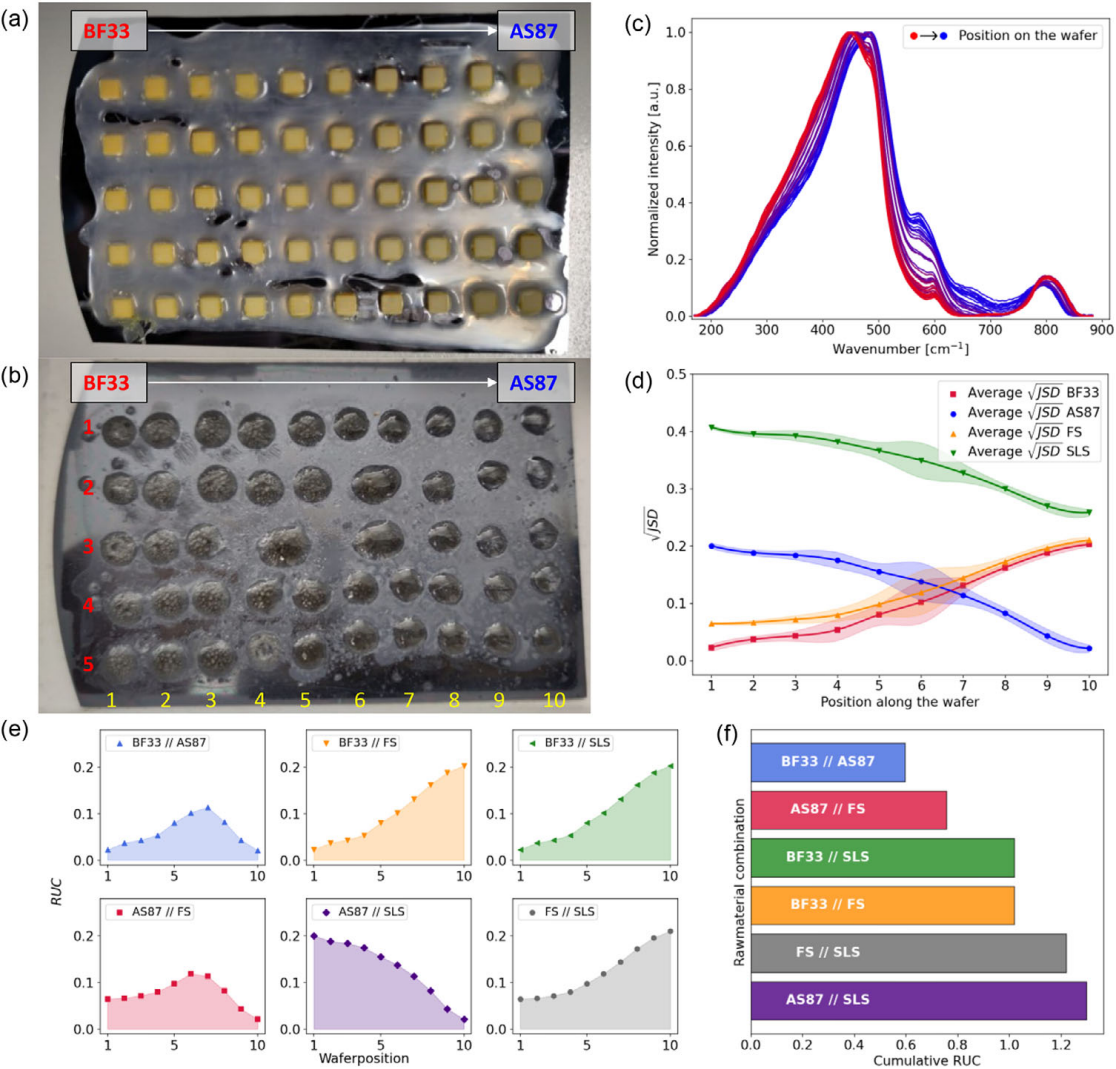
(a) Printed, and subsequently (b) melted specimens comprising incremental mixtures of BF33 and AS87, supported on a silicon wafer. (c) Normalized Raman spectra of the printed, then melted specimens. (d) Average column‐by‐column RSI values considering mixtures with four selected references: BF33, AS87, fused silica (FS), and soda‐lime silicate (SLS)—with error margins calculated through error propagation. (e) Residual uncertainty (RUC) of each raw material combination (excluding self‐combinations). (f) Cumulative RUC of each raw material combination. Similarity maps referencing to manually mixed samples are provided in Figure S4.

Subsequently, building upon the validated printing quality and the established Raman data‐processing platform, the HTS approach is now extended to a larger experimental scale. In this stage, fifty specimens—again consisting of incremental mixtures of BF33 and AS87 glasses with five replicates per composition—were printed and subsequently melted, as shown in Figure 4a,b. As observed previously, all samples remain transparent and exhibit no evidence of crystallization or phase separation. However, because melting was conducted on a supporting substrate rather than under free‐standing conditions, the resulting sample appearances vary across the compositional series. Specifically, (i) BF33‐rich compositions, characterized by higher viscosity [28], displayed a greater prevalence of inhomogeneities (i.e., bubbles), whereas (ii) AS87‐rich formulations exhibited signs of cracking due to their higher coefficient of thermal expansion (CTE, with values for BF33, AS87, and Si being 3.3·10^−6^, 8.7·10^−6^, and 2.6·10^−6^ K^−1^, respectively) [28, 29]. Additionally, because of their proximity and surface tension, six specimens merged during melting (Figure 4b) and were therefore excluded from further analysis.

Despite these material considerations, such effects do not hinder HTS evaluation via Raman spectroscopy, thereby enabling the method to be applied to a wide range of glass matrices regardless of their—often unknown—thermomechanical properties. As such, the Raman spectra of all specimens are presented in Figure 4c. The analysis focuses on the 170 – 875 cm^−1^ spectral region, where systematic changes can be observed and baseline removal performs well. Correspondingly, the average $\sqrt{JSD}$ (denoted Raman similarity index (RSI) in the following) values of each column are illustrated in Figure 4d, considering mixtures with four representative matrices of glass science—fused silica (FS) and soda‐lime silicate (SLS), in addition to BF33 and AS87 (Figure S3)—as a means to evaluate the glass chemical composition against a reference spectral catalog. Positions 1–6 show the best match with BF33 while steadily increasing in their distance, with a maximum at position 6 just before the best matching glass changes to AS87. The certainty of classification in the middle of the wafer suffers mainly due to the exclusion of the merged samples and because the glass structure starts to change significantly. The distances in the first 6 wafer positions to the FS are relatively low, indicating that there could be a decent present and unsurprisingly both BF33 and FS have a low modifier content and exhibit similar vibrational modes in the selected region. By intuition, the SLS would be the worst match as a possible base material candidate due to its high modifier content and lack of any intermediates like Al or B. This is confirmed by Figure 4d where the SLS is in every case the worst match possible.

To further elucidate this intrinsic case and, more broadly, to establish a quantitative and operator‐independent analysis metric for HTS‐based glass compositional identification using an expanded library of representative glass matrices and corresponding Raman spectra, the residual uncertainty (RUC) was adopted as a decisive parameter. The RUC, extracted at each wafer position, is defined as:

(3) $$RUC=Min\;\left[\sqrt{JSD_{n}}\;\left(C_{1}\right);\sqrt{JSD_{n}}\;\left(C_{2}\right)\right]$$

where C_1_ and C_2_ denote the initial raw materials, and $\sqrt{JSD_{n}}$ represents the average $\sqrt{JSD}$ value at wafer position n; the cumulative RUC, subsequently used to determine the numerically best‐matching glass combination, is calculated as:

(4) $$\mathrm{cumulative}\;\mathrm{RUC}=\sum_{n=1}^{N}RUC_{n}$$

where N corresponds to the number of columns on the wafer. In the present case study, the derived RUC and cumulative RUC values are illustrated in Figure 4e,f, respectively. In such two‐component systems, a valid match can only be considered when the candidate combination converges toward near‐zero RUC values at both compositional extremes (0 and 100 wt.%). Among the six possible combinations of the four selected representative glasses (excluding self‐combinations), only two satisfy this criterion: BF33 // AS87 and FS // AS87. Notably, for a linear mixture of two glasses, the maximum RUC values occur in‐between these compositional extremes, corresponding to the region of greatest dissimilarity relative to the raw materials. In a final step, discrimination between the two remaining candidates is simply achieved using the cumulative RUC metric, which clearly favors the BF33 // AS87 combination over FS // AS87 (0.598 and 0.759, respectively). This highlights the potential and suitability of the present HTS platform—combining day‐scale synthesis with automated Raman spectral processing and quantitative, operator‐independent evaluation—for large‐scale materials discovery (∼100 samples per day).

### 2.4 Beyond Raman Spectroscopy and Glass Discovery

**FIGURE 5:**
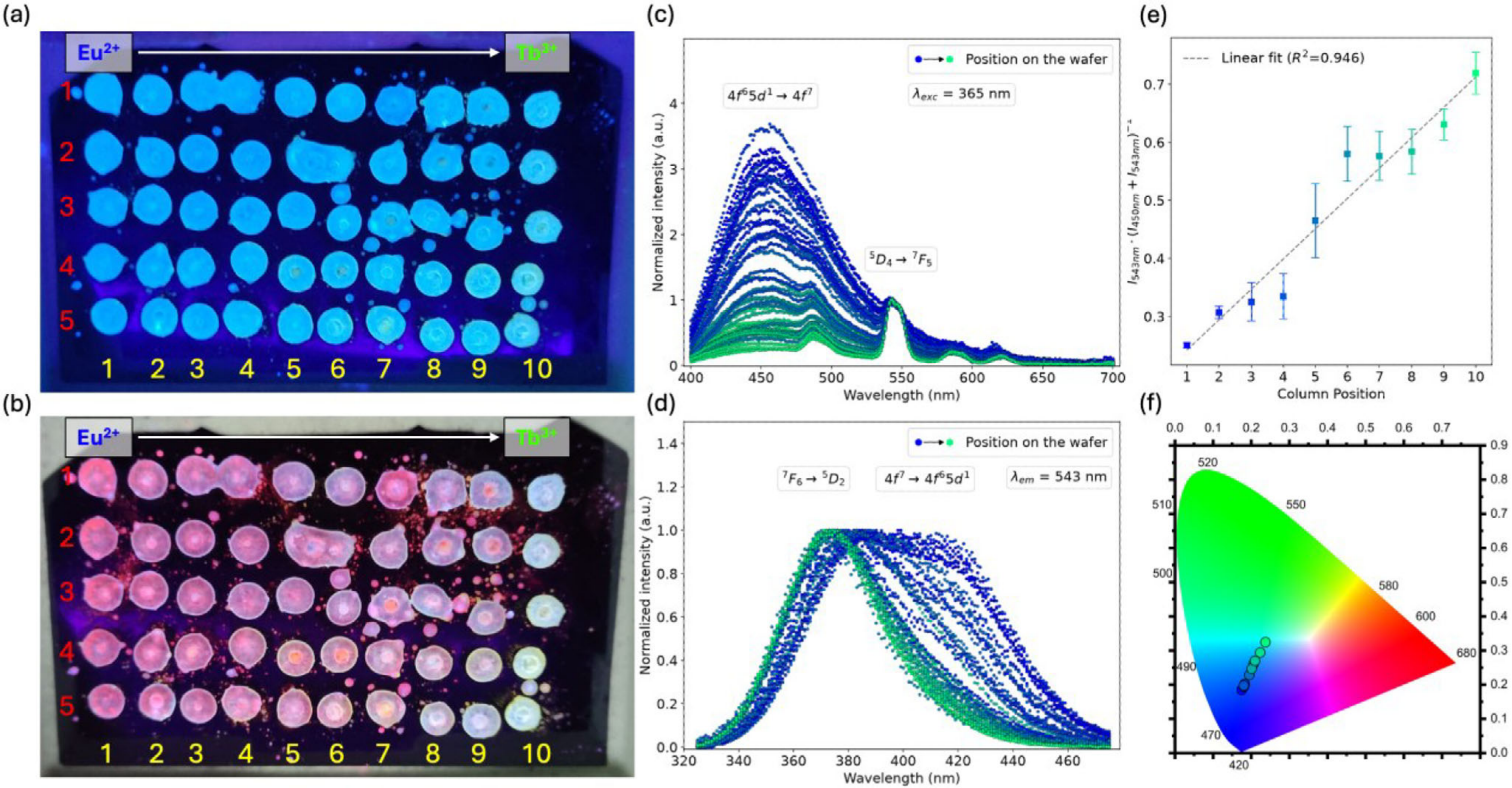
(a) Printed, and subsequently melted specimens comprising incremental mixtures of BF33 doped with Eu2O3 and Tb2O3 supported on a silicon wafer under 365 nm UV‐light, (b) under 254 nm UV‐light. (c) Emission spectra of the specimens normalized toward the 543 nm Tb3+ emission peak. (d) Normalized excitation spectra for the 543 nm emission peak. (e) The Intensity ratio averages of the 543 nm Tb^3+ 5^D_4_→^7^F_5_ emission and the 450 nm Eu^2+^ 4f^6^ 5d^1^→4f^7^ emission inside the standard errors including a linear fit with R^2^ = 0.946. (f) CIE coordinates of the emission spectra.

As an additional proof of concept, another 50‐specimen series was printed using two BF33 slurries, one doped with 3 wt.% Eu_2_O_3_ and the other with 2 wt.% Tb_2_O_3_.These were mixed in the same incremental way as the specimens from Figure 4a,b but ranging from 0 wt.% Tb‐dopant to 100 wt.%. The wafer resulting after thermal processing is shown in Figure 5a,b under two different wavelengths. A clear gradient in photoluminescence color is visible, corresponding to the variation in dopant concentration.

The emission spectra shown in Figure 5c show a clear positional dependence. The broad emission of Eu^2+^ at approx. 450 nm [30] dominates the spectrum on column position 1 of the wafer and progressively loses in intensity toward position 10 while the peak of the Tb^3+^ at 543 nm [31] emission grows in relative intensity. Small amounts of Eu^2+^/Tb^3+^ emissions are still visible at positions 1 and 10 due to the above‐mentioned minimal slurry cross contamination and the merged sample at position row = 2 columns = 5 and 6 was excluded for consistency. As a result, the emission color under 365 nm light gradually shifts, as Eu is substituted by Tb, from deep blue to greenish‐blue (CIE coordinates x, y of 0.17, 0.18 and 0.24, 0.32, respectively shown in Figure 5f). The excitation spectra in Figure 5d show a similar trend where the broad Eu^2+^ excitation transitions into a localized Tb^3+^ excitation at 370 nm.

By calculating the intensity ratio of the Eu^2+^/Tb^3+^ emissions shown in Figure 5e the visual transition of the previous figures is confirmed. The linear fit exhibits a high R^2^‐value which confirms that most of the variation in the intensity ratio is mainly caused by the position on the wafer itself.

This further illustrates the versatility and utility of the slurry based HTS approach, in case, for studying dopant effects. In addition, the fluorescence measurements show that the presented HTS approach is not purely limited to be evaluated by Raman spectroscopy and is compatible with more HTS compatible characterization techniques.

## 3 Discussion

In this study, a versatile HTS platform was developed to accelerate glass discovery. The approach first consisted of delineating the key parameters, from batch preparation, slurry formulation, and printing parameterization, enabling reliable large‐ and day‐scale 3D printing of cm^3^‐scale specimens. After melting and characterization, the resulting glasses exhibited chemical and thermal properties comparable to those obtained by conventional synthesis routes, thereby validating the applicability of the HTS methodology.

Subsequently, an automated workflow for Raman spectral processing and evaluation was implemented and applied to these systems. This workflow enabled rapid, preparation‐free sample assessment while maintaining a level of analytical efficiency comparable to established techniques such as DSC and EDS.

Finally, the complete HTS platform—encompassing both synthesis and characterization—was scaled to a representative case study involving fifty individual samples. By selecting appropriate supports and heating conditions, the workflow demonstrated its robustness and capacity to accelerate the exploration of chemically diverse glass compositions simply based on libraries of representative glass matrices and associated Raman spectral fingerprints.

Overall, the proposed strategy provides a systematic and operator‐independent framework for comparing glass systems under controlled conditions, highlighting its relevance for both academic and industrial research. As a proof of concept, the platform was applied to rare‐earth‐doped photoluminescent glass hosts, where the rapid yet reproducible evaluation of their behavior illustrates the potential of the approach for identifying compositions capable of delivering advanced material performance whilst addressing sustainability considerations.

## 4 Methods

### 4.1 Sample Preparation

To evaluate both the printing quality and the high‐throughput capability of the developed process, four glass series were designed. Series 1 and 2 each comprised eleven specimens with nominal compositions corresponding to mixtures of BF33 and AS87 in 10 wt.% increments, prepared either by conventional glass synthesis (Series 1) or by the high‐throughput process (Series 2). Series 3 consisted of fifty specimens with nominal compositions of BF33‐AS87 mixtures in 11.1 wt.% increments, each composition produced in five replicates using the high‐throughput process. Series 4 included fifty specimens with nominal compositions corresponding to mixtures of Eu_2_O_3_‐doped BF33 (3 wt.%) and Tb_2_O_3_‐doped BF33 (2 wt.%) in 11.1 wt.% increments, again with five replicates per composition, produced using the high‐throughput process.

To this end, the raw materials employed for glass synthesis included borosilicate glass (BF33) and aluminosilicate glass (AS87) obtained from Schott AG, Fused Silica (FS) was obtained from Heraeus and Soda lime silicate (SLS) obtained from DWK Life Sciences. Eu_2_O_3_ (Projector, 99.999%) and Tb_2_O_3_ (Projector, 99.999%). Samples produced by conventional glass synthesis were prepared by weighing and homogenizing the glass powders, followed by melting in a Pt crucible at 1500°C for 2 h, quenching, and annealing at 500°C for 2 h. The resulting bulk glass samples were subsequently cut and polished to optical‐grade quality and stored prior to further characterization. The preparation of printed glasses—including batch preparation, slurry formulation and calibration, printing parameterization, and final melting—is described step‐by‐step in the following section.

### 4.2 Printing Process

The reference bulk glasses were first crushed by ball milling in a ZrO_2_‐lined container together with 15 ZrO_2_ balls (20 mm in diameter) at 300 rpm for 30 min to obtain a coarse glass powder (∼100 g). The resulting powder (∼23 g) was then transferred to another ZrO_2_‐lined container together with ethanol (twice the powder mass) and 260 g of ZrO_2_ balls (2 mm in diameter) and milled at 380 rpm for 45 min. The obtained fine powder was dried overnight at 120°C, subsequently sieved to achieve a particle size of smaller 40 µm, and dried a final time overnight at 400°C to remove residual solvent and adsorbed water.

The resin solution consisted of two monomers: 80 wt.% trimethylolpropane ethoxylate triacrylate (TMPETA, purity > 99%) and 20 wt.% 2‐hydroxyethyl‐methacrylate (HEMA, purity > 99%). Two photoinitiators, camphorquinone (CQ, purity > 97%) and ethyl 4‐(dimethylamino)benzoate (purity > 99%), were added at 1.6 wt.% each relative to the monomer mixture. Sudan Orange G (dye content = 85%) was incorporated at 0.1 wt.% to produce a deep orange solution, thereby preventing overcuring. All chemicals were purchased at Sigma Aldrich. The resin formulation was designed to polymerize under blue light irradiation.

The fine, dried glass powder was subsequently mixed with the resin solution and a dispersing agent (DISPRBYK‐180 purity > 99.99% BYK‐Chemie GmbH). To enhance dispersion quality, an initial mixture consisting of 50 wt.% glass powder, 40 wt.% resin solution, and 10 wt.% dispersing agent was prepared in a ZrO_2_‐lined container together with 15 ZrO_2_ balls (10 mm in diameter) and milled at 600 rpm for 10 min. In a second step, additional portions of glass powder and resin solution were introduced using the same milling procedure. The final composition consisted of 61.7 wt.% glass powder, 33.3 wt.% resin solution, and 5 wt.% dispersing agent. The resulting slurry was further milled for 18 h at 600 rpm (10 min milling followed by 20 min rest cycles to prevent overheating) to ensure complete homogenization.

A Lithoz CeraFab 2M30 multicomponent 3D printer was used for stereolithographic printing. The printer consists of a light source with an x‐y resolution of 35 µm, a build platform with a z‐axis resolution of 10 µm, two movable slurry tanks (designated primary or secondary depending on the slurry), a cleaning station equipped with a rolling paper towel, and a touchscreen interface for operation. Printing can be performed either in alternating or inline mode.

The exposure energy (mJ·cm^−2^) and irradiance (mW·cm^−2^) of the lamp can be controlled independently, with the exposure time determined by their ratio. The following exposure conditions were used: 600 mJ·cm^−2^ and 40 mW·cm^−2^ (BF33 slurry); 1800 mJ·cm^−2^ and 40 mW·cm^−2^ (AS87 slurry); 750 mJ·cm^−2^ and 40 mW·cm^−2^ (Eu_2_O_3_‐doped BF33 slurry); 900 mJ·cm^−2^ and 40 mW·cm^−2^ (Tb_2_O_3_‐doped BF33 slurry). For all specimens, ten adhesion layers were first printed at twice the baseline lamp energy (double the time) to ensure sufficient adhesion to the build platform. In a second step, support structures were printed using the primary slurry under baseline conditions to elevate the samples above the platform, thereby preventing contact between the plate and the slurry contained in the tanks and avoiding cross‐contamination or slurry loss. Finally, parallelepiped‐shaped specimens of the desired dimensions (shapes and volumes) were printed atop these supports, resulting in a total printing time of ∼8 h for reference studies. Instead of aiming for intricate 3D structure, multicomponent deposition was used to optimize admixture toward obtaining homogeneous glass sample during the subsequent melting step. For this, random pixel patters as well as layer‐by‐layer printing was considered, in which pixel or layer fractions were adapted to the fractions of admixed chemical compounds (see Figure S6). All glasses produced in this work were printed using the layer‐by‐layer printing strategy.

Following printing, specimens from Series 2 were removed from the support structures, washed in di(propylene glycol) methyl ether (DPM) to remove residual slurry, and the remaining supports were polished off. For Series 3 and 4, the specimens remained attached to the build plate and were repeatedly dipped in DPM, cured under a blue light lamp for 30 min, and subsequently hot‐glued onto a heated silicon wafer. After polymerization of the adhesive, all specimens were simultaneously detached from the plate using a sharp tip, and the residual supports were polished off. Free‐standing printed specimens (Series 2) were placed in alumina crucibles and heated for two days at 500°C (heating rate: 0.165 K·min^−1^) under a fume hood to remove organic components. Supported specimens (Series 3 and 4) were treated under identical debinding conditions. Debinding was employed to protect the platinum sheets from corrosion and can be avoided for the wafer melting, however, was as a process step for consistency. The Series 2 samples (80–100 mg) were then transferred into platinum foil, heated to 1500°C (22.5 K·min^−1^) for 2 h, and quenched on a copper plate prior to polishing. Supported specimens (Series 3 and 4) were first heated to 1350°C (22.5 K·min^−1^) and subsequently heated slowly to 1400°C (2.0 K·min^−1^) for 2 h to minimize temperature overshoot before quenching on an alumina brick. This approach prevented oxidation of the silicon wafer, which was initially selected due to its affordability and favorable thermal and chemical stability; however, this could be avoided entirely by employing an alternative wafer material.

### 4.3 Characterization

The elemental composition (±1 atomic%) of each sample was analyzed using a TESCAN MIRA field emission scanning electron microscope (FE‐SEM) equipped with a Schottky field emission cathode and an integrated TESCAN EDS (energy‐dispersive X‐ray spectroscopy) detector. Elemental analysis and EDS mapping were performed at an accelerating voltage of 20 kV, with a working distance of approximately 15 mm under high‐vacuum. Prior to measurement, all samples were coated with a thin gold layer (15 nm) using a sputter coater (Leica EM ACE600). Analytical uncertainties (e.g., for the Al/(Si+Al) atomic ratio) were calculated from error propagation.

Differential scanning calorimetry coupled with thermogravimetric analysis (DSC‐TGA) experiments were conducted using STA 449 F1 Jupiter from Netsch. Each sample was loaded in a platinum crucible, and heated with a 10 K·min^−1^ heating rate from 20 up to 750°C under N_2_ flow.

Florescence spectra were collected via a florescence microscope. The excitation source is a Xenon‐lamp. Emission spectra were collected between 400 – 700 nm using a 365 nm excitation wavelength. Excitation spectra were collected between 325 – 475 nm at 543 nm emission. All measurements use an integration time of 0.1 s and a monochromator bandwidth of 5 nm. The light was focused on the cusp of the sample before each measurement using the microscope with a similar setup to Figure S5. The measurement of the entire 5 × 10 wafer takes below 2 h.

Unpolarized Raman spectra were acquired with a confocal Raman microscope (Renishaw InVia) equipped with a 2400 lines·mm^−1^ diffraction grating and a 514 nm diode laser operating at 50 mW. The laser power was set to 100% and the spectra were collected between 100–1460 cm^−1^. For each sample a 10 s measurement time and 3 accumulations were performed resulting in 30 s measurement time per spectrum. For the specimens on the wafer, the microscope was manually focused on a defect free position on the cusp of the specimen as shown in Figure S5. The measurement of the entire 5 × 10 wafer takes below 2 h and can potentially be automated for further acceleration of the HTS.

### 4.4 Numerical Methods

#### 4.4.1 Preprocessing

After the Raman spectrum is collected a few numerical steps are necessary to ensure that the comparison via the $\sqrt{\mathrm{JSD}}$ can be done reliably and without artifacts.

The first step is to detect and remove any cosmic rays that occur due to random chance. Cosmic rays are defined by a sharp and sudden increase in intensity which makes them easily detectable via the modified z‐score of the derivative of the spectrum [32]. The detected spike is masked and then interpolated via a Whittaker‐Eliers smoother [33] which usually results in smoother interpolations than linear or polynomial methods. The interpolation scheme is constrained by a consideration of the sum of the second derivative of the spectrum. If the interpolation increases this metric, it is discarded. This algorithm allows for aggressive yet precise automated cosmic ray removal (crr) with no loss in spectral quality. After crr is performed, the spectrum is cropped and smoothed with a savgol filter with half‐window size = 5 and polyorder = 3. After cropping and smoothing, a baseline is subtracted. The manually polished samples from series 1 & 2 just need a simple—min value correction due to their optimal shape, surface quality and no carrier material. The samples on the wafer require a rubberband baseline correction and thus a smaller chunk of the spectrum is analyzed to minimize numerical artifacts. Afterward the spectrum is area normalized and is ready for numerical analysis.

#### 4.4.2 Distance Calculation

Before the $\sqrt{\mathrm{JSD}}$ is calculated the spectra are convolved with a Blackman window of half size = 5 to create a weighted average at wavenumber position x. This further limits the influence of the noise. Second the spectra are checked if they are aligned properly and exhibit the same number of values. A difference vector is calculated from the wavenumber arrays of both spectra used for comparison. If the maximum offset of any wavenumber pair exceeds the spectral resolution of the grating, the comparison is stopped. Same happens if one spectrum contains more measurement points than the other. For distance calculation, only the intensity vectors are needed. Every sample spectrum is compared against all selected reference spectra, and the result is saved in a n x m matrix which is written to an excel‐file. All parameters used in the preprocessing step and the alignment statistics are saved as well.

## Author Contributions

L.W. conceived the idea and provided funding and resources. I.S.‐G. and J.S. prepared the slurries and the raw materials. I.S.‐G. and J.S. designed the printing process. I.S.‐G. printed, melted, and polished all glasses. I.S.‐G. designed, validated, and applied the Raman‐based processing platform with input from N.M.A.K. F.S. performed EDS measurements. I.S.‐G. and A.D. performed and evaluated the florescence measurements. I.S.‐G., A.D., and L.W. analyzed and interpreted the data and wrote and revised the manuscript. All authors added and commented on the manuscript and its revisions.

## Conflicts of Interest

The authors declare no conflicts of interest.

## Supporting information




**Supporting File**: advs77590‐sup‐0001‐SuppMat.docx.

## Data Availability

The data that support the findings of this study are available from the corresponding author upon reasonable request.
