# Supplementary material for: High‐Throughput Screening for Accelerated Oxide Glass Discovery
Source: Adv Sci (Weinh). 2026 Sep 6:e77590. Online ahead of print. doi: 10.1002/advs.77590 (PMC13547689; doi:10.1002/advs.77590)
Supplement: Supplementary file 1 [file ADVS-9999-e77590-s001.docx]

**Supporting Information**

**High-Throughput Screening for
Accelerated Oxide Glass Discovery**

Igor Seibel-Geraschenko^1^, Josef Slowik^1^, Franziska Scheffler^1^, Alexis Duval^1^, N. M. Anoop Krishnan^2^, and Lothar Wondraczek^1,3,*^

*^1^Otto Schott Institute of Materials Research, Friedrich Schiller University Jena, Lessingstraße 14, 07743 Jena, Germany*

*^2^Department of Civil Engineering, Indian Institute of Technology Delhi, Hauz Khas, New Delhi, 110016, India*

*^3^Center for Energy and Environmental Chemistry (CEEC), Friedrich Schiller University Jena, Philosophenweg 7a, 07743 Jena, Germany*

*^3^*

Table of Contents

This document contains

- Supplementary Figures S1 to S7
- Supplementary Table S1

**Figure S1.** Chemical analysis (SEM-EDX) of 3D-printed glass samples after melting. Top: sample micrographs, shown by way of example for 50/50 (wt%) admixture of BF33 and AS87 (a), pure AS87 (b) and pure BF33 (c). The lines mark the positions of EDX linescans, as shown in the corresponding chemical profiles (bottom, (d-f), respectively).

**Figure S2.** Self-similarity of reference spectra used for sample classification. The heatmap shows the Raman classification of manually mixed reference samples through the $\sqrt{JSD}$ parameter obtained by self-referencing. Blue colors indicate regions with little divergence among the reference spectra, in particular, when the fraction of AS87 is high.

**Figure S3.** Reference Raman spectra of pure endmember glasses as used for Jensen-Shannon divergence calculations and automated sample classification. Spectra are preprocessed is indicated in Figure 3(a) of the main text.

**Figure S4.** Wafer-based high-throughput classification of glass samples by Raman spectroscopy. (a) Preprocessed Raman spectra of manually created reference glasses from BF33/AS87 admixture, using the same thermal protocol as applied for the samples in Figure 4(b). (b) Corresponding $\sqrt{JSD}$ heatmap of reference materials in comparison with the Raman spectra of the 3D printed specimen from Figure 4(c).

**Figure S5.** Three distinct measurement positions (a-c) showing the microscope positions on the selected specimen, (d-f) shows the corresponding Raman laser spot on the surface at reduced intensity, (g) shows the raw spectra corresponding to the measurement position and (h) shows the processed spectra as described in the main manuscript.

**Figure S6**. Print schemes for efficient premixing. Top: layer-by-layer approach (a) under the microscope on a 3D printed BF33/AS87 50/50 wt% sample and (b) schematic representation of alternating slurry layers. Bottom: random pattern approach, in which the composition of the mixture is translated into pixel fractions, (c) microscope image of 3D printed BF33/AS87 50/50 wt% sample, and (d) schematic representation of pixel pattern.

**Figure S7:** Merging experiment of manually placed specimen on a wafer where the surface was roughened using a 360-grade polishing paper with (a) the specimen after melting, and (b) and (c) measurements of distances between closely positioned specimens.

**Table S1.** Atomic composition of the samples shown in Figure S1 (in at.%). Nb and Zr are given as one, given the limitations of EDS analysis.

| Sample | Si | O | Al | Na | K | Mg | Nb/Zr |
| --- | --- | --- | --- | --- | --- | --- | --- |
| 3D-printed 50/50 (wt%) admixture | 33 | 55 | 5 | 5 | 1 | 1 | 1 |
| pure AS87 | 26 | 53 | 8 | 7 | 3 | 2 | 1 |
| pure BF33 | 36 | 59 | 2 | 3 | - | - | 1 |
